# Consent in organ transplantation: putting legal obligations and guidelines into practice

**DOI:** 10.1186/s12910-022-00791-y

**Published:** 2022-07-05

**Authors:** Farrah Raza, James Neuberger

**Affiliations:** 1 Pembroke College, University of Oxford, St. Aldates, Oxford, OX1 1DW UK; 2 Max Planck Institute for Social Anthropology, Advokatenweg 36, 06114 Halle, Germany; 3 Liver Unit, Queen Elizabeth Hospital, Birmingham, B15 2TH UK

**Keywords:** Organ donation, Organ transplantation, Consent, Legal requirements

## Abstract

Consent in medical practice is a process riddled with layers of complexities. To some extent, this is inevitable given that different medical conditions raise different sets of issues for doctors and patients. Informed consent and risk assessment are highly significant public health issues that have become even more prominent during the course of the Covid-19 pandemic. In this article we identity relevant factors for clinicians to consider when ensuring consent for solid organ transplantation. Consent to undergo solid organ transplantation is more complex than most surgical and other clinical interventions because of the many factors involved, the complexity of the options and the need to balance competing risks. We first out the context in which consent is given by the patient. We then outline the legal principles pertaining to consent in medical practice as it applies in the UK and the implication of recent legal judgments. The third section highlights specific complexities of consent in organ transplantation and identifies relevant factors in determining consent for organ transplantation. The fourth section offers practical recommendations. We propose a novel ‘multi-factor approach’ to informed consent in transplantation which includes understanding risk, effective communication, and robust review processes. Whilst understanding risk and communication are a given, our suggestion is that including review processes into the consent process is essential. By this we specifically mean identifying and creating room for *discretion* in decision-making to better ensure that informed consent is given in practice. Discretion implies that health care professionals use their judgement to use the legal judgements as guidance rather than prescriptive. Discretion is further defined by identifying the relevant options and scope of clinical and personal factors in specified transplantation decisions. In particular, we also highlight the need to pay attention to the institutional dimension in the consent process. To that end, our recommendations identify a gap in the current approaches to consent. The identification of areas of discretion in decision-making processes is essential for determining when patients need to be involved. In other words, clinicians and healthcare professionals need to consider carefully when there is room for direction and where there is little or no room for exercising discretion. In sum, our proposed approach is a modest contribution to the on-going debate about consent in medicine.

## Contextualising consent in solid organ transplantation

As with any form of therapeutic intervention, it is accepted that the patient should give appropriately informed consent before solid organ transplantation. However, the challenges of organ transplantation are far greater than for most other medical or surgical interventions. Transplantation is usually offered to increase either length or quality of life or both. In most cases, the patient will be anxious and their ability to receive, understand and process information may be affected by either the organ failure itself (such as encephalopathy in those with advanced renal or liver disease or hypoxia consequent on heart or lung disease) or by medication required to treat the patient, or both. Encephalopathy may not always be clinically apparent and may fluctuate in severity over time. This will add to the challenges of ensuring the patient is fully informed and can make their own decision. The amount of information that the patient needs to be fully informed is vast and exceeds that required for most medical and surgical interventions. In addition to understanding the implications of not proceeding with transplantation and the implications of the transplant procedures and its consequences (as outlined in more detail below), the patient has to be aware of the short and longer term consequences of transplantation, including the increased risk of death or graft failure from immune mediated damage, infection, de novo malignancies or cardiovascular disease, the need for life-long immunosuppression (for the majority of recipients), the inherent consequences of immunosuppression and need for monitoring and some limitations on life-style [1]. Furthermore, the candidate needs to be aware of the risks associated with the donor organ and the deciding which organs are acceptable. For example, the patient may have to decide whether to take accept an offered organ and risk possible fatal graft failure or donor transmitted disease or whether to decline the organ and risk death or serious illness which precludes transplantation, while awaiting another offer. This challenge is exacerbated by the clinical and scientific difficulties in defining risk and the unpredictability of when a suitable but lower risk-associated organ will be available [2]. Whereas in those with liver or lung failure, there is no appropriate long-term life support, in those with kidney failure, organ support is available as renal dialysis and all forms of renal support (such as haemodialysis or peritoneal dialysis) carry their own associated risks and benefits [3]. To add to the complexity, these decisions often have to be made in the small hours of the night as that is when most organ offers are made [4]. Studies suggest a variable understanding of risks associated with higher risk organs [5]. Risks of transplantation, non-transplantation and outcomes vary between organs. For example, if a kidney graft fails, the patient can return to dialysis until another organ becomes available over the ensuring months or years, although they will have undergone a procedure which proved unsuccessful and made subsequent transplants more hazardous because of the effects of sensitisation and previous surgery. In contrast, if a heart, lung or liver graft fails, unless a suitable graft can be found immediately, death will follow shortly after the onset of organ failure.

In many instances, the data on which to base decisions are limited, not least because data based on historical data obviate the impact of developments, both positive (such as improved immunosuppression) and negative (such as the donor pool is limited and a higher frequency of high-risk organs).

Before discussion of how the patient can be informed and valid consent given in solid organ transplantation, it is worth pointing out that, in the UK and some other jurisdictions, for organ donation by deceased donors, consent may be deemed, and the donor has not actively given consent [6]. In this context, we use the term ‘deemed consent’ to mean consent is given even though the person has not actively given or indicated consent; this is in contrast to implied or non-verbal consent where an action by the person implies consent (such as offering their arm for venesection). Furthermore, where consent is expressly given, the consent for organ donation remains valid unless it is withdrawn, and donation usually takes place be many years after consent was first given. The law on donating organs in Wales changed in 2013 with the enactment of The Human Transplantation (Wales) Act 2013 which introduced in law the concept of deemed consent for deceased organ donation and other UK nations have followed (albeit with some variation) [7] These changes in the consent system have reignited debates about the nature of consent and how valid consent can be secured safely and efficiently. Thus, there is a range of conceptions of consent and this paper focusses on consent to solid organ transplantation and highlights the specific challenges in this context.

## Consent to medical treatment

This section sets out some of the key conceptualisations of, and principles governing, consent to medical treatment in law with the purpose of outlining key issues with consent before turning to solid organ transplantation in section three. According to Beauchamp and Childress, there are four fundamental principles of medical ethics which include: beneficence, nonmaleficence, autonomy, and justice [8]. Within this framework, consent is a central aspect of medical ethics and has continued significance in contexts where notions of individual autonomy are highly valued. Respect for patient autonomy has become one of the markers of a good standard of care. The process of giving and obtaining consent is based on ethical practice, supported by local, national and international guidelines, underpinned by a legal framework.

However, it is important to note that the principle of consent is understood and conceptualised in the medical context in a myriad of ways ranging from: informed, deemed or broad. Informed consent requires the patient to be given and understand *adequate information about the risks* of a particular course of action. Deemed consent assumes that a patient agrees to a certain course of action *unless* they explicitly or implicitly express otherwise. Rebutting the presumption in favour of consent, often requires evidence. Broad consent allows for a *range* of activities to be carried out under a generic ‘yes’ or agreement to a particular study or treatment [9]. Although the notion of broad consent is useful, it has limitations in some contexts, such as in the context of health data platforms where consent to a particular trial might not necessarily cover future (re-)use of patient data [10]. In addition, the Human Tissue Act 2004 uses the term ‘appropriate consent’. Appropriate consent defines the standard of consent with regards to the particular circumstances. The variations of consent have led some commentators to argue that consent ‘now takes a bewildering variety of forms’ [11].

Each conceptualisation of consent raises different sets of legal, ethical, and practical issues. The validity of consent might, therefore, depend on how consent is conceptualised. With each conceptualisation of consent, questions of adequate information and capacity arise. A patient must have capacity and be provided with adequate information about potential side effects of a particular treatment option. A related issue is one of communication and evidence. Communication is a two-way process: just as the health care professionals need to provide all relevant information in a manner that is understandable by the patient with capacity, the patient has a responsibility to take note of the information provided and seek clarification where needed. Therefore, any notion of consent needs to factor in communication of information and risk disclosure as we outline in section four.

It is well established that consent is a process [12] and that evidence of a signed consent form does not count as conclusive evidence of valid consent [13]. The General Medical Council’s (GMC) most recent *Guidance on Consent* [12] emphasises that whilst consent forms can be useful for record keeping, ‘filling in a consent form is not a substitute for a meaningful dialogue tailored to the individual patient’s needs’. So, when is consent meaningful? What does it entail? And how should the requirements of valid consent be tailored to meet the needs of patients in need of a transplant?

The principle of consent can be broken down to include various parts of the consent process and into various legal obligations. Without valid consent, a doctor is liable to have committed battery or breached their duty of care. The ingredients of ‘valid’ consent include at least the following:

(i) *Capacity:* as defined in law (for example, in England and Wales this is covered by the Mental Capacity Act (2005) with similar acts in other jurisdictions)
(ii) *Voluntariness:* freedom from coercion or undue influence
(iii) *Choice:* understanding the availability of therapeutic options so allowing meaningful choice
(iv) *Disclosure of material risks* and other relevant information

However, each one of these requirements are disputed and have been subject to litigation. In recent years, the legal, professional and ethical principles underlying the obtaining and giving of consent has undergone significant changes. Since the leading judgment from the UK Supreme Court in *Montgomery (Appellant) v Lanarkshire Health Board (Respondent) (Scotland)* a patient’s autonomy and beliefs are considered to be central to medical decision-making [14]. Patients should be thought of as ‘co-decision makers’ and not as passive receivers of care and medical advice. *Montgomery* is hailed as repudiating the ‘paternalistic’ doctor-patient relationship, and how these principles should be incorporated into practice is discussed below.

*Montgomery* concerned a pregnant diabetic woman whose baby was more likely to have shoulder dystocia. The risk of a baby developing this condition was calculated to be approximately 9–10%. The consultant obstetrician, in charge of Ms Montgomery’s case did not discuss the specific risks associated with shoulder dystocia, although she accepted the risk of shoulder dystocia constituted a ‘high risk’. Ms Montgomery’s baby did suffer severe disabilities as a result of complications suffered during vaginal delivery. Ms Montgomery brought two claims of negligence. The first claim concerned the assertion that she should have been informed about the risk of shoulder dystocia and the option of delivery by elective caesarean section. The second issue concerned the management of labour. The Courts considered several complex issues including the disclosure of risk and causation. Here causation is used in the legal sense that if the patient *had* been aware of the risks, she would have made a different decision [15]. Ms Montgomery failed on causation at first instance and on appeal. However, the Supreme Court accepted that Mrs Montgomery would have been probably have elected to be delivered of her baby by caesarean section had she been informed of the risk of shoulder dystocia. The Supreme Court unanimously held that the obstetrician’s failure to disclosure of all material risks amounted to a breach of the Consultant’s duty of care. The duty of risk disclosure was set out clearly at paragraph 87:

An adult person of sound mind is entitled to decide which, if any, of the available forms of treatment to undergo, and her consent must be obtained before treatment interfering with her bodily integrity is undertaken. The doctor is therefore under a duty to take reasonable care to ensure that the patient is aware of *any material risks* involved in any recommended treatment, and of *any reasonable alternative* or variant treatments. (our emphasis)

A narrow exception to this duty exists. Notably, the duty of disclosure does not require disclosure of *all* risks. How ‘materiality’ is to be understood is further defined at para. 87:

The test of materiality is whether, in the circumstances of the particular case, a reasonable person in the patient’s position would be likely to attach significance to the risk, or the doctor is or should reasonably be aware that the particular patient would be likely to attach significance to it.

Thus, a risk might be material in two ways where (i) *a reasonable person* in the patient’s position would be likely to attach significance to the risk *or* if (ii) the doctor is or should reasonably be aware that *the particular patient* would be likely to attach significance to it. Material risk is not conclusively defined with reference to percentages, but percentages can be indicative where the risk does not concern a borderline assessment; such an example given in *Tasmin v Barts Health Trust*, where a risk of 1 in 1000 might safely be considered to be non-material [16]. Post-operative risks should also be disclosed [17]. The Supreme Court in *Montgomery* provided further guidance on how to interpret the duty of disclosure at paras. 89–91 [14]. In particular, the Court held that the significance/materiality of a risk depends on a ‘magnitude of factors including the nature of the risk and its effects on the patient’ and that the doctor’s advisory role involves ‘dialogue’. Moreover, Lady Hale emphasised that ‘It is now well recognised that the interest which the law of negligence protects is a person’s interest in their own physical and psychiatric integrity, an important feature of which is their autonomy, their freedom to decide what shall and shall not be done with their body’. She also stated that ‘… it is not possible to consider a particular medical procedure in isolation from its alternatives. Most decisions about medical care are not simple yes/no answers. There are choices to be made, arguments for and against each of the options to be considered, and sufficient information must be given so that this can be done…’ The Supreme Court in *Montgomery* developed the law in a more positively patient-centred direction and rejected the earlier approach to information disclosure as articulated in *Sidaway v Board of Governors of the Bethlem Royal Hospital* [18]. Their Lordships differed as to the standard of care regarding risk disclosure and negligence. Prior to *Montgomery*, there was a debate about the standard of care concerning information disclosure and doctor’s liability regarding how much information a patient should be given. In *Sidaway* the claimant argued that she had not been informed about a small risk of damage to her spinal column arising from her operation. The Court held that the failure to warn her of the risk was not negligent. Lord Diplock held that the ‘Bolam test’ applies to all aspects of a doctor’s duty of care. Whereas, Lord Bridge proposed a modified version of the ‘Bolam test’. In *Bolamv* *Friern Hospital Management Committee*[19] the court held that ‘[a] doctor is not guilty of negligence if he has acted in accordance with a practice accepted as proper by a responsible body of medical men skilled in that particular art’.

Responses to the *Montgomery* judgment have been varied. Some hail the judgment as constituting a significant shift in the way in which the doctor-patient relationship is to be understood. Emily Jackson [20] attributes the importance of the judgment to the ‘wholesale rejection of the reasonable doctor test and its adoption instead of the partnership model of medical decision-making embodied in GMC's Guidance [12]. Jonathan Herring et al. [21] argue that:

the shift marked by Montgomery in the basis of duty of care is a shift in underpinning values: it is a shift from the clinician’s interpretation about what would be best for patients to the values of (to what is significant or matters from the perspective of) the particular patient concerned in the decision in question. But the values of the particular patient do not thereby become paramount. The Montgomery test of duty of care requires the values of the particular patient to be balanced alongside the values of a reasonable person in the patient’s position.

Others adopt a more cautious approach and argue that ‘the reality is that Montgomery will make little difference to healthcare practice and consent in the UK…The UK Supreme Court endorsed a view of consent most lawyers, and doctors thought already prevailed, and largely reflects UK GMC's Guidance on the issue’ [22]. The GMC’s 2008 Guidance was acknowledged as highly influential, but nevertheless, the Supreme Court held that it ‘was necessary to impose legal obligations, so that even those doctors who have less skill or inclination for communication, or who are more hurried, are obliged to pause and engage in the discussion which the law requires’. The GMC subsequently updated its Guidance post-Montgomery. However, Emily Jackson [20] argues that the modern healthcare system is increasingly impersonal, and as such, doctors cannot be expected to know in advance what matters to individual patients. This is why we propose a multi-factor approach in section four to take into account the changes.

The *Montgomery* judgment unambiguously rejected the paternalistic model of the doctor-patient relationship in favour of a more patient-centred, co-operative model of decision-making. Subsequent case law emphasises that dialogue should be considered a necessary part of the informed consent process [23]. Moreover, dialogue should be adequate. In *Thefaut v Johnston* the Court emphasised that ‘the issue is not so much the means of communication but its adequacy’ [24].

The courts have post-*Montgomery* acknowledged that the test of materiality is a mixture of the objective and subjective. However, there is continuing uncertainty about ‘the actual extent to which subjective factors relating to the actual patient are relevant since the greater degree of subjectivity inserted into the assessment the further one departs from the standard of the reasonable patient’. The decisions of the Courts are not intended to act as a substitute for decision of the decision-maker. Accordingly, it is necessary to turn to professional guidelines on consent for further advice on how to interpret the numerous obligations that arise from the consent process. The General Medical Council’s most recent guidelines outline seven key principles of decision making and consent. These include the need to involve patients in decision making and the need for dialogue and for relaying information in an accessible manner. Principle 4 [12] sets out:

Doctors must try to find out what matters to patients so they can share relevant information about the benefits and harms of proposed options and reasonable alternatives, including the option to take no action.

The GMC’s guidance sets out how doctors should disclose risks with both objective and subjective dimensions in mind:

22. It wouldn’t be reasonable to share every possible risk of harm, potential complication or side effect. Instead, you should tailor the discussion to each individual patient, guided by what matters to them, and share information in a way they can understand.
23. (a) Recognised risks of harm that you believe anyone in the patient’s position would want to know. You’ll know these already from your professional knowledge and experience.
24. (b) The effect of the patient’s individual clinical circumstances on the probability of a benefit or harm occurring.
25. (c) Risks of harm and potential benefits that the patient would consider significant for any reason.

The Guidance further elaborates [12] that the scope of the consent must be clear:

You must be clear about the scope of decisions so that patients understand exactly what they are consenting to. You must not exceed the scope of a patient’s consent, except in an emergency.

Moreover, the Human Tissue Authority’s most recent guidance on Consent [25] sets out:

*Valid consent*

40. For consent to be valid it must be given voluntarily, by an appropriately informed person who has the capacity to agree to the activity in question. The person should understand what the activity involves, any reasonable or variant treatment and, where appropriate, what the material risks are. The test of materiality is ‘whether, in the circumstances of the particular case, a reasonable person in the patient’s position would be likely to attach significance to the risk, or the doctor is or should reasonably be aware that the particular patient would be likely to attach a significance to it’.

In sum, the guidelines require doctors and healthcare professionals to consider the general material risks of a particular treatment, patient specific risks, and the desires, alternatives and values of patients, and the quality and form of communication necessary to achieve a dialogue in which the patient is autonomous and informed.

Informed consent, however, has its limitations. Jessica Berg and colleagues [26] identify four limitations. Firstly, informed consent does not *in and of itself* create opportunities when choices are lacking. Secondly, informed consent relies on the existence of a cultural context that facilitates a certain kind of decision-making process or mode of communication. Thirdly, Berg and colleagues argue that ‘informed consent may lose its impact when the parties to it lack a common interpretative framework, for example, if they approach the decision-making context with different conceptions of illness and its treatment’. Finally, they argue that interpersonal communication is not necessarily improved by disclosing all the facts. If informed consent is, at its core, aimed at protecting individual autonomy then the limitations of the notion of autonomy should also be taken into account. Autonomy is arguably limited in the following ways. Individual autonomy is commonly shaped by interpersonal relationships and recent literature increasingly focusses on ‘relational autonomy’. The family structure, or lack thereof, might influence which and how decisions are made [27]. An individual’s cultural and religious beliefs or practices might limit the options available to them. Moreover, autonomy is limited where an individual lacks capacity, in the legal sense, to make autonomous decisions (as outlined in the Mental Capacity Act). The ability to understand certain sets of complex information might also be limited by a range of social factors. For example, a lack of medical training inevitably means that a certain level of knowledge is simply inaccessible to the non-clinician/specialist*.* These factors might be relevant to clinicians when engaging in the consent process with their patients. Limitations to informed consent does not mean that legal duties can be ignored. Informed consent is a necessary but not always a sufficient condition for ensuring a patient’s best interests are realised. As acknowledged by scholars drawing on socio-legal methodological approaches to consent, ‘Concepts, and especially open-textured evaluative concepts such as ‘autonomy’ and ‘consent’, acquire much of their content and evaluative significance from the social and institutional environments in which they function’ [28]. We argue that a multi-factor approach to informed consent helps to ensure it is achievable in practice. Moreover, our approach includes ‘robust review’ as a relevant factor that takes into account the institutional dimension.

Although there have been many cases in UK law based around the issue of consent to medical interventions, we are aware of only two cases involving consent in organ transplantation. One case relates to the rights of parents to decline organ transplantation for the child: the child was dying of end-stage liver disease and, although the clinicians agreed that transplant was the best option, the parents felt that this was not in the child’s interest. The parents’ views were upheld [29]. In the other case [30], a surgeon burned his initials on the liver graft. It was accepted that what the surgeon did was calculatedly harmless and no physical damage beyond the 'transient and trifling' was done. However, this was a criminal, non-consensual physical interference. The surgeon’s action was not included in the patient’s consent.

The responsibilities of the patient are less clearly defined. For example, the NHS Constitution [31] lists the responsibilities of the patient, and these include statement that the patient should recognise that they can make a significant contribution to their own and their family’s good health and wellbeing, and take personal responsibility for it. Other sources are more proscriptive as regards consent. Thus, Findlaw [32] states ‘although a doctor is required to inform their patient about benefits, risks, and alternative treatments, patients must also play a part in the informed consent process. Patients must listen to the physician and should ask questions if they don't understand or would like more detailed information’. Others have concluded [33] a patient is not free to receive treatment voluntarily without knowledgeably authorizing it. Thus, while few national guidelines or legal precedents explicitly state this, we do believe that the patient does share the responsibility for informed consent.

## Identifying relevant risk factors in giving and determining consent in transplantation

There are various complex risks that arise from organ transplantation that are not seen in most surgical interventions. A risk assessment will involve both standardised and personalised factors. There are several broad categories of risk [1, 2, 34, 35]:

- Risks associated with remaining with non-transplant support
- Risks associated with the surgery (short and long-term) and anaesthesia
- Risk associated with graft failure (both short and long-term)
- Risks associated with the donor (such as transmission of infection or malignancy which may be known or unintentional)
- Risks associated with the organ (such as organs from donors after circulatory death or obese donors that are more likely to fail
- Risks associated with immunosuppression (which are both drug-specific and generic (such as increased risk of some cancers, some infections, cardiovascular and renal toxicity))
- Change in lifestyle (such as need for long-term care, avoidance of unnecessary risk, avoidance of smoking, possible teratogenicity, need for lifelong (usually) immunosuppression)
- Risks of recurrent disease and early and late rejection

Some of the risks can be readily easily quantified and are based on historical data, although, as in other areas of clinical practice, advances in treatments and changing donor and recipient demographics mean that such historical data may no longer be accurate. Furthermore, the number of low frequency but serious side-effects in organ transplantation is enormous. For example, for the most commonly used long-term immunosuppressive agent (Tacrolimus) the Patient Information Leaflet lists 63 side-effects of the drug (specific 13, 6 very common, 15 common, 13 uncommon, rare 10, very rare 5, unknown 1) [36]. Add to that the many possible adverse events, the amount of information becomes unmanageable and uninterpretable for most candidates. Public trust in information provided by nurses or doctors is relatively high [37].

Furthermore, to give appropriately informed consent the patient must be able to balance risks. Understanding of the balance of risks is highly complex and many of those factors cannot be reasonably assessed with confidence [38–40]. For example, a patient with progressive advanced cirrhosis will have a given life expectancy with medical treatment and an estimated survival after transplantation. There are several prognostic models that will generate survival probabilities, but these have wide confidence intervals. Similar considerations apply deciding whether to accept an organ from a higher risk donor (such as an obese donor or one with a history of cancer) or decline that offer and wait for another lower risk organ. In addition, such issues involve delicate ethical dilemmas that might create additional burdens for patients and doctors alike. In most other areas of clinical practice, the responsibility of the clinician is to the individual patient: because of organ shortage, the clinician is usually looking after several patients who are all potential candidate for the life-saving organ. For many patients, especially heart, lung or liver recipients, these decisions will be literally a matter of life or death. Therefore, risk disclosure in transplantation is complex because risk assessment is challenging because obtaining valid consent must be occur alongside the balancing of other competing factors.

The reality is that clinical decision-making is subject to context and constraints such as the skills of the clinician and time available for discussion [41]. Furthermore, the patient who is generally has a limited life-expectancy without transplant or is on renal support, is not only stressed and affected by the disease process but their mental capacity affected by the disease process or its treatment. Thus, there is a huge amount of information that the patient must be given and understand if meaningful and valid informed consent is to be given. Understanding will depend on many factors including social and cultural beliefs, understanding, and cognitive ability. Studies in other clinical situations have demonstrated a huge variety in the level of understanding of risks and the additional complexity of organ transplantation place additional burdens on the potential transplant candidate [42]. In these circumstances, how can clinicians ensure that a patient has understood the significant (on going) risks and made the right decision for them?

## Practical recommendations: proposing a multi-factor approach

In light of the above, how should clinicians inform potential transplant candidates in a meaningful manner so that the patient not only makes the decision that is right for them at the time or listing and immediately prior to transplantation? Some guidance on provision of information is already given by professional bodies and much information is available provided by national bodies (such as NHS Choices), professional bodies and the transplant units. Furthermore, the practice in transplant units is information is given over time, by several health professionals (including specialist nurses, doctors, pharmacists) and using a variety of formats. However, personal clinical/practical experience reinforces the finding that patient understanding, and recollection is limited. We suggest a ‘multi-factor approach’ based on three key over-arching themes that include: (a) understanding risk, (ii) effective communication, and (iii) robust review processes will help to ensure that informed consent is achieved in practice at each relevant stage of the consent process. Therefore, we recommend that clinicians consider the following practical issues when advising and informing patients who are in need of an organ transplant. Our multi-factor approach does not seek to supplant existing guidelines on consent, but rather acts as additional practical guidance. Our approach is both under and over-inclusive as compared to the GMC guidelines [12] on consent. In particular, the focus on review processes distinguishes our approach from some of the existing guidelines since we emphasise that the institutional processes of review are a fundamental part of the consent process: risk assessment and effective communication can only be fully realised provided there are institutional and process-based mechanisms in place that facilitate and guarantee informed consent. Moreover, review processes are particularly important in the context of organ transplantation since various ethical issues do arise. Thus, review processes should be considered substantive obligations in that the transplantation centre monitors its consent processes. Whilst the GMC guidelines lay down seven core principles, our approach fine tunes that by proposing a multi-factor approach.

### Understanding risk

Understanding risk is priority since it is an integral part of informed consent as demonstrated by the case law. Consent can only be informed once patients have been informed of the risks of a particular procedure or course of treatment. It is obvious that the identification of risks from a clinical viewpoint is fundamental and measured in accordance with clinical standards, and risks are presented in a way that a patient can understand. What often makes risk identification complicated is how it is understood by patients and their families. *Montgomery* [14] makes it clear that risk also needs to be ‘personalised’. That is to say, not only does the objective identification of risk matter, but the subjective perception of risks matter because this influences patient’s decision-making. Identifying and discussing risks can be complex, emotional, and time consuming. Freeman points out that knowledge of a fact or percentage is not to be equated with understanding [43]. It is, therefore, useful to break this particular requirement of consent down into a series of specific practical points.:

(i) In face-to-face discussions, clinicians should focus on the material risks. While selection of these need to be tested in collaboration with patient groups, we suggest these include as a minimum:
  - survival and death rates after transplantation
  - alternative therapies and their risks and benefits and implications; this option should include palliative care where appropriate
  - types of donors and the various classes of donor organs and their associated risks
  - major causes and timing of graft loss and death post-transplant
  - life-style issues post-transplant (such as need for life-long immunosuppression, compliance, alcohol and drug use, implications for fertility)
(ii) The patient and their family members should be told of the existence of less common risks. Less common risks should then be given in supplementary information which are accessible both in terms of format and language.
(iii) The personalisation of risk and subjective assessment of risk must take into account:
  - What is important to the patient and their family or loved ones
  - To what extent/how the proposed course of treatment will impact the patient’s lifestyle and quality of life etc.
  - How the patient feels about the relevant consideration in their case.

### Effective communication

Effective communication is essential since without the proper communication of different aspects of medical treatment, it will be difficult to determine the actual wishes of the patient. Therefore, effective communication is also central to informed consent. The question remains what counts as ‘effective’ in a given situation. There are no robust guidelines that can definitively offer answers for each case. It must also be stressed that communication is a two-way process. All ‘parties’ must be seen as active participants and not passive recipients of information. However, there are a number of practical considerations that ought to be taken into account:

(iv) Health care professionals should be empowered to give advice to help the transplant candidate decide their preferred option. This might mean, for example, that advice is given at the appropriate level: more complex information might need to be communicated by the clinician or senior healthcare professional.
(v) Evidence: documentation or evidence of imparting relevant information: records of discussions, details of information given (written and other) is important to ensure that doctors and other health care professionals involved in giving information and helping ensure consent is informed routinely discharge their duties to inform patients
(vi) Clinicians should understand which donor risks the recipient is prepared to accept by investigating whether any specific clinical or social factors impact the patient’s decision
(vii) Personalisation of risks relevant to that person: the mode of delivery is key here. This means that in addition to an appropriate risk assessment and understanding (see above), the mode of delivery also matters. This approach is in line with ‘dialogue’ approach set out in *Montgomery* [14]

### Robust review

Finally, it is important that clinicians, healthcare professionals and transplantation centres have review procedures to ensure consistency and dialogue. As we have outlined, some of the ambiguities and complexities of realising valid consent in practice concern the scope of the doctor’s risk assessment, risk disclosure, and assessment of personal factors in decision-making. Robust review includes considerations as relevant to the perspective of the patient, medical staff, and transplantation centre. We suggest that the identification of discretion in decision-making processes will help to understand when and how patients can be better engaged in the consent process. By the term discretion, we mean that health care professionals should be able, and indeed encouraged, to use their own expertise and judgement to tailor the information provided to the needs of the individual. We therefore imply that the legal judgements should be seen as guidance rather than be proscriptive. More specifically, within each centre, discretion exists at several points during the consent process. It is important that discretion is thought about more explicitly: because it is precisely where clinicians and healthcare professionals have room to choose between different courses of actions, that they should engage patients. The approach has to be adapted not only to the patient, but in accordance with the specific transplant centre in order to take in to account the institutional dimension. As with the patient giving consent in other areas, where possible the patient should have time to receive and consider the information given in a format that is understandable to the patient, have opportunities to seek further information and opportunities to change their mind. Listing for transplantation usually takes place several weeks, months or even years before transplantation, so consent should be reaffirmed every 6 months and again immediately before transplantation. As the patient’s condition progresses, the balance of risks may change so the patient may wish to receive a higher risk graft. Robust review, then, includes the following factors:

(viii) Identification of discretion at different points of the consent process and understanding how this might differ depending on categories of patients or the organ.
(vi) Taking into account the specific institutional dimensions relevant to the consent process namely:
  - the specific internal processes
  - staff training: format and frequency
  - feedback from relevant actors
  - data collection on the above.

The above should not be considered as merely a ‘proceduralised box-ticking exercise’, but rather seen in the context of consent as a process based on good communication and dialogue. Asking the patient questions such as ‘what matters to you most?’ [44] might help to understand the patient’s priorities. The health care professional should also take steps to ensure that the transplant candidate does understand the issues surrounding the decision. To realise the above recommendations in practice, it is necessary to implement relevant processes, provide training and resources to ensure that the conditions necessary for satisfying informed consent are achievable in busy clinics with all the time pressures involved. Although review is important in evaluating patient’s understanding, many studies have suggested that recollection is often low even when written information is provided [45–47].

## Conclusion

Informed consent is a core legal and ethical obligation during the course of any medical treatment. *Montgomery* emphasises the need to ensure that patients are involved in a dialogue and understand the material risks of various treatment options. As we outline, transplantation presents unique challenges. We have argued that a multi-factor approach can help to ensure informed consent. Understanding risk is essential to risk disclosure and dialogue. Moreover, robust review processes mean that doctors need to identify the areas of discretion in their decision-making processes and subject these/this to internal review either through peer review or by alternative means. In other words, the consent process as envisaged by *Montgomery* [18], is strengthened once doctors and health care professionals implement internal systems that facilitate a more comprehensive form of consent. Indeed, suggestions of harnessing digital tools [48] to enhance patient-centred decision making, do not necessarily resolve the substantive problems and complexities with valid consent. Our proposal is a modest contribution to the on-going debates about optimising conditions in the clinic in order to achieve fully informed and therefore valid consent.

## Data Availability

Not applicable.
